# Delayed perinephric hematoma following ESWL in an anticoagulated patient: A case report

**DOI:** 10.1016/j.ijscr.2025.111626

**Published:** 2025-07-07

**Authors:** Aseel Eid, Ammar Hassouneh, Salsabeel Bishawi, Aya Milhem, Jana Dibas, Sondos Baradia

**Affiliations:** a Department of Medicine, An Najah National University, Nablus, Palestine; b Department of Radiology, Hebron Governmental Hospital, Hebron, Palestine; c Department of Medicine, Hebron University, Hebron, Palestine

**Keywords:** Extracorporeal shock wave lithotripsy, Perinephric hematoma, Anticoagulation, Nephrolithiasis, Conservative management, Case report

## Abstract

**Introduction and importance:**

Extracorporeal shock wave lithotripsy (ESWL) is a widely used non-invasive treatment for renal stones; however, it carries a risk of hemorrhagic complications, particularly in patients with comorbidities such as hypertension, diabetes, and anticoagulation therapy. Perinephric hematoma is a rare but potentially serious complication that requires prompt recognition and appropriate management.

**Case presentation:**

We report a case of a 58-year-old male with hypertension, diabetes, and long-term anticoagulation who developed a massive perinephric hematoma one week after undergoing ESWL for bilateral renal stones. He presented with severe left flank pain and a significant hemoglobin drop, with imaging confirming a large perinephric hematoma. He was managed conservatively in the intensive care unit (ICU) with close monitoring, bed rest, fluid resuscitation, pain control, and temporary cessation of anticoagulation. Over 10 days, his condition improved without the need for invasive intervention, and he was discharged on oral medications with a planned follow-up for renal function assessment and anticoagulation resumption.

**Clinical discussion:**

This case highlights the importance of early diagnosis using CT imaging in patients presenting with post-ESWL flank pain and anemia. Conservative management, including strict monitoring, supportive care, and temporary anticoagulation withdrawal, was successful in achieving hemodynamic stability without invasive intervention. This underscores the need for individualized treatment strategies based on patient risk factors and clinical presentation.

**Conclusion:**

This case underscores the importance of early recognition of ESWL-associated hemorrhagic complications, particularly in high-risk patients. It also highlights the effectiveness of conservative management as a viable approach to achieving successful recovery without the need for surgical or interventional procedures.

## Highlights

• ESWL is effective for kidney stones but may cause rare hemorrhagic complications such as perinephric hematoma.
• High-risk patients (e.g., with hypertension, diabetes, and on anticoagulants) are more vulnerable post-ESWL.
• We report a 58-year-old male who developed a large perinephric hematoma one week after ESWL.
• The patient was managed conservatively with ICU monitoring and temporary anticoagulation withdrawal.
• Conservative treatment was successful, avoiding surgery, and the patient was discharged with follow-up care.

## 1 Introduction

Deposition of inorganic elements and organic compounds together in the kidneys leads to a condition named as nephrolithiasis [1]. It is a widespread urological condition, presented as sudden onset colicky flank pain, nausea, and vomiting [2]. Kidney stone treatment varies depending on several factors; therefore, many medical techniques are available ranging from the most invasive one which is open surgery to non-invasive extracorporeal shock wave lithotripsy (ESWL) [3].

ESWL is a pioneering procedure used to fragment urolithiasis by applying shockwaves from outside the body and making them smaller pieces to facilitate its passage from the body [4]. Even though it is a safe process, naturally its effectiveness is accompanied by certain side effects and complications [5]. The most common sign after the procedure is hematuria, due to shock wave induced injury. Whereas subcapsular, intrarenal, and perinephric hematoma develop in 1 % of patients. In most cases, perinephric hematomas are small and resolve spontaneously without medical intervention [6]. The occurrence of these complications depends on several factors. These factors generally classified into stone-related, patient-related, treatment-related factors. Patient related factors include hypertension, older age, and anticoagulant medication consumption. Moreover, the characteristics of the stone like location, dimensions, and solidity have been detected. In addition, factors related to treatment such as total energy [7].

## 2 Case presentation

A 58-year-old male with a history of hypertension, diabetes mellitus, and prior cardiac catheterization, who was on long-term anticoagulation therapy, presented with severe left-sided flank pain one week after undergoing extracorporeal shock wave lithotripsy (ESWL) for bilateral renal stones. A pre-procedural non-contrast CT scan had revealed a 1.6 × 1.2 cm calculus in the left lower calyx and a 1.5 × 0.6 cm stone in the right kidney.

The patient initially developed severe left flank pain one day prior to presentation and sought medical attention at the emergency department, where he was treated with analgesia. His symptoms temporarily improved, and he was discharged. However, the pain persisted and worsened over the following day, prompting his return to the emergency department.

On arrival, the patient appeared to be in significant pain but was alert and oriented. He was hemodynamically stable and afebrile. Physical examination revealed a soft, non-distended abdomen with significant left renal angle tenderness. A Foley catheter was inserted for urinary monitoring. Laboratory tests showed a marked drop in hemoglobin from 15.3 g/dL one week prior to 10.5 g/dL at presentation, with a hematocrit of 33 %. Creatinine was 1 mg/dL, and urinalysis showed 30–35 red blood cells per high-power field.

**Fig. 1:**
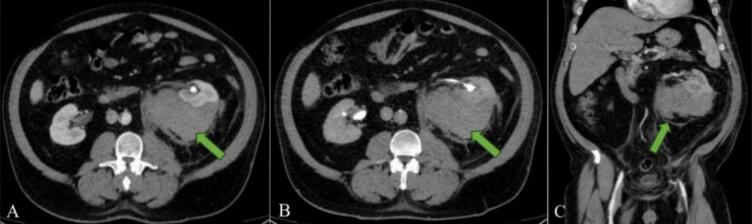
Contrast-enhanced abdominal CT scan demonstrating a large left perinephric hematoma (green arrow) exerting a mass effect on the left kidney. The delayed phase (B) reveals anterior displacement of the left kidney and pelvicalyceal system, with no evidence of contrast extravasation. (For interpretation of the references to colour in this figure legend, the reader is referred to the web version of this article.)

A contrast-enhanced CT scan of the abdomen and pelvis revealed a large left perinephric hematoma causing a mass effect on the left kidney. The delayed phase showed anterior displacement of the kidney and pelvicalyceal system, but no active contrast extravasation, suggesting a contained hemorrhage without ongoing bleeding (Shown in Fig. 1). Given the significant hemoglobin drop, the presence of a large perinephric hematoma, and the patient's anticoagulated status, he was admitted to the intensive care unit for close monitoring and supportive management.

In the ICU, he was placed on strict bed rest with hemodynamic monitoring. He was kept nil per os to minimize gastrointestinal workload, and diabetic control was managed through a sliding scale insulin regimen with regular blood glucose monitoring. He received intravenous fluids with 3000 mL of normal saline over 24 h, along with IV paracetamol for pain control, IV pantoprazole for gastroprotection, and IV cefepime for empiric antibiotic coverage. Serial complete blood counts were performed every 6 h to monitor hemoglobin levels, and strict vital sign monitoring was maintained. Anticoagulation therapy was withheld during the ICU admission to prevent further bleeding.

During the first week of ICU admission, serial hemoglobin monitoring showed stabilization without further significant drops. The patient remained hemodynamically stable, with no signs of active bleeding. By day 10, his clinical status had improved significantly. His pain was well-controlled, and repeat laboratory investigations showed stable hemoglobin levels. Renal function remained preserved, and follow-up imaging confirmed that the perinephric hematoma was resolving without further expansion.

After 10 days in the ICU, he was transitioned to oral medications and prepared for discharge. At the time of discharge, he was hemodynamically stable, pain-free, and able to tolerate a soft diabetic diet. He was prescribed Evox 500 mg once daily and acetaminophen 500 mg four times daily for pain management. He was scheduled for follow-up in the urology clinic to assess hematoma resolution and renal function, with a nephrology consultation to evaluate renal function and adjust anticoagulation therapy. Gradual resumption of anticoagulation was planned based on hematologic and renal assessment.

## 3 Discussion

Extracorporeal shock wave lithotripsy (ESWL) was first introduced in the 1980s as a non-invasive technique for fragmenting renal calculi [8]. Since its inception, it has become the preferred modality for managing kidney and ureteral stones due to its minimally invasive nature and efficacy, providing a viable alternative to surgical interventions such as percutaneous nephrolithotomy (PCNL) and ureteroscopy (URS) [4,5].

The management of urinary calculi encompasses both conservative and surgical approaches. Conservative management may involve observation, supported by medical expulsive therapy (MET), whereas surgical intervention is indicated in cases where conservative management fails after four to six weeks, the patient experiences recurrent renal colic necessitating multiple emergency department visits, or when complications such as urinary tract infection, sepsis, or renal function deterioration arise [9]. Among the available surgical options, ESWL remains the treatment of choice for renal stones ≤20 mm (non-lower pole) and lower pole stones ≤10 mm, as well as for mid or distal ureteral stones in patients who decline URS. In pediatric patients, ESWL is recommended as a first-line approach for renal calculi ≤20 mm and as a secondary option for ureteral stones that fail MET [9]. However, for pediatric cases with renal calculi >20 mm, ESWL and PCNL are both acceptable options, depending on anatomical considerations.

Despite its advantages, ESWL is not without complications. While generally well-tolerated, potential adverse events include hematuria, ureteral obstruction, infection, renal impairment, and hematoma formation [4], with the known complications summarized in (Table 1).

**Table 1:** Complications of extracorporeal shock wave lithotripsy (ESWL).

Complication	Description
Flank pain	A common, unwanted side effect that patients often experience. May occasionally lead them to request the discontinuation of the treatment. Often Controlled with analgesics [4]. Worsening or persistent pain requires further investigation [10].
Hematuria	An expected outcome, it is believed to result from trauma to the renal parenchyma and vasculature. In most cases, it is self-limiting and typically resolves within 12 h. However, if hematuria persists or worsens, it may indicate substantial renal injury and warrants further clinical investigation [9].
Hematoma	Can be perinephric, subcapsular, or intranephric. An infrequent yet potentially fatal complication. The primary clinical manifestations include severe flank pain and hematuria. Significant predisposing factors for perirenal hematoma include a history of hypertension, an increased body mass index (BMI), and use of anticoagulation medications [4,11].
Infection	Including bacteriuria, urosepsis, perinephric abscess formation, and endocarditis. Rarely leads to death. ESWL-induced renal trauma and vascular disruption can facilitate bacterial entry into the bloodstream. Destruction of infected calculi also releases bacteria into the urine, which may be systemically absorbed [12].
Gastrointestinal lesions	A study demonstrated gastroduodenal erosions in 80 % of patients undergoing both pre- and post-ESWL endoscopic evaluations. The underlying mechanism remains unclear, although lesions tend to be more prevalent in patients treated in the prone position or those who receive shock waves exceeding the recommended dosage [5].
Contusion	Involves skin contusion and bruising, in addition to renal contusions which are treated conservatively [4].
Ureteral blockage	Incomplete fragmentation can cause ureteral obstruction (steinstrasse) in about 3 % of ESWL cases, with 6 % requiring surgery. Treatment is usually conservative, but may include a double J stent, ureteroscopy, or repeat ESWL [4].
Cardiovascular complications	The association between ESWL and hypertension remains controversial. However, diastolic hypertension has been documented to increase following ESWL in multiple studies, with one suggesting being dose-dependent, with a greater number of shock waves correlating with more severe diastolic hypertension [12]. Arrhythmia may also occur, with an incidence rate of 11–59 %. Most of which are premature ventricular contractions [5]. Abdominal aneurysms leakage or rupture [4] has also been reported.

To understand the pathophysiology of hematoma formation post-ESWL, it is essential to examine the underlying mechanism of action. The procedure employs high-energy shock waves, which are focused on renal calculi to induce fragmentation through multiple physical forces, including compressive stress, cavitation, shear stress, cleavage, and spall fracturing. The energy dissipation at the stone-fluid interface facilitates effective stone disintegration, allowing the fragmented pieces to be expelled via the urinary tract [10]. The shock waves propagate through the body with minimal energy loss due to the similar acoustic impedance of soft tissues, thus reducing the risk of significant injury to surrounding structures. However, the high-density difference between calculi and surrounding fluid causes energy dissipation at the stone-fluid interface, enhancing fragmentation but also posing a risk of collateral tissue damage. Advanced imaging techniques such as ultrasound or fluoroscopy are utilized to ensure accurate targeting and minimize off-target effects [4,10].

Despite these precautions, renal and perinephric hemorrhagic complications can still occur, primarily due to vascular injury. The most frequent hemorrhagic complication is hematoma formation, which may be perinephric, subcapsular, or intrarenal [6]. The reported incidence of perinephric hematoma following ESWL varies significantly depending on the imaging modality used for detection. It ranges from 0.1 to 0.6 % when assessed by ultrasonography, whereas MRI or CT studies reveal an incidence as high as 20–25 %. Most hematomas are small and self-limiting, resolving over six weeks to six months without significant morbidity [6,11,12]. However, in rare instances—accounting for <1 % of cases—hematomas can be massive, leading to life-threatening hemorrhage and hemodynamic instability, necessitating urgent intervention [13].

Several risk factors predispose patients to hematoma formation post-ESWL, including hypertension, increased body mass index (BMI), and anticoagulant or antiplatelet therapy [4,14]. The clinical presentation varies, with symptoms ranging from flank pain, gross hematuria, and ileus to complete asymptomatic presentations [6,15]. While mild post-procedural pain is common and generally resolves with conservative analgesia, severe pain refractory to treatment should raise suspicion for a significant underlying complication, warranting further imaging with contrast-enhanced CT. Similarly, hematuria following ESWL is typically benign, but persistent or worsening hematuria should prompt further evaluation to exclude significant hemorrhagic complications [16,17].

Perinephric hematoma, although rare, represents a serious post-ESWL complication. While many cases resolve spontaneously, life-threatening hematomas necessitate prompt resuscitative measures and hemodynamic stabilization. A study analyzing 3620 ESWL procedures reported the development of 24 hematomas, with one-third of affected patients requiring blood transfusions due to severe hemorrhage. The study also highlighted hypertension and anticoagulant use as key predisposing factors for hematoma formation [16,17]. In severe cases, interventional radiology techniques such as selective arterial embolization may be required to control bleeding while preserving renal function. Early recognition and a tailored management approach are critical in mitigating the morbidity and potential mortality associated with this rare yet severe complication.

This case report aims to highlight the risk of delayed massive perinephric hematoma following extracorporeal shock wave lithotripsy (ESWL), particularly in high-risk patients with comorbidities such as hypertension, diabetes, and anticoagulation use. By presenting this case, we emphasize the importance of early recognition, appropriate imaging, and individualized management strategies to prevent complications and ensure patient safety. Our report underscores the role of conservative management in achieving hemodynamic stability and successful recovery without the need for invasive intervention.

The patient was managed conservatively in the intensive care unit with strict hemodynamic monitoring, bed rest, fluid resuscitation, pain control, and temporary cessation of anticoagulation therapy. Supportive care, including intravenous fluids, analgesia, and prophylactic antibiotics, helped stabilize his condition. Serial hemoglobin monitoring showed no further significant drops, and follow-up imaging confirmed gradual hematoma resolution. After 10 days of hospitalization, he was discharged in stable condition with planned nephrology and urology follow-ups for renal function assessment and anticoagulation resumption.

## 4 Conclusion

This case underscores the risk of massive perinephric hematoma as a delayed complication of ESWL, particularly in patients with predisposing factors such as anticoagulation use, hypertension, and diabetes. While ESWL remains a widely used, minimally invasive treatment for nephrolithiasis, clinicians must be vigilant for potential hemorrhagic complications. Conservative management, including hemodynamic monitoring, supportive care, and withholding anticoagulation, proved effective in this case, avoiding the need for invasive intervention. Timely recognition and close follow-up are crucial to ensuring patient safety and optimal renal function recovery. Future studies should explore risk stratification models to identify patients at high risk for post-ESWL hematoma formation, allowing for personalized management strategies.

## 5 Methods

This work has been reported with the SCARE criteria.

## Author contributions

All Authors contributed equally to the report (reviewing the literature, writing the case presentation, and discussing the case information). All authors reviewed the results and approved the final version of the manuscript.

## Consent

Written informed consent was obtained from the patient for publication of this case report and accompanying images. A copy of the written consent is available for review by the Editor-in-Chief of this journal on request

## Ethics approval

Our institution does not require ethical approval for reporting individual cases or case series.

## Guarantor

Dr. Aseel Eid, Dr. Ammar Hassouneh, and Dr. Salsabeel Bishawi accept full responsibility for the integrity of the study, had access to the data, and controlled the decision to publish.

## Funding sources

No funding sources.

## Declaration of competing interest

The authors state that they have no conflict of interest to be mentioned.
